# PET CT Identifies Reactivation Risk in Cynomolgus Macaques with Latent *M*. *tuberculosis*

**DOI:** 10.1371/journal.ppat.1005739

**Published:** 2016-07-05

**Authors:** Philana Ling Lin, Pauline Maiello, Hannah P. Gideon, M. Teresa Coleman, Anthony M. Cadena, Mark A. Rodgers, Robert Gregg, Melanie O’Malley, Jaime Tomko, Daniel Fillmore, L. James Frye, Tara Rutledge, Robert M. DiFazio, Christopher Janssen, Edwin Klein, Peter L. Andersen, Sarah M. Fortune, JoAnne L. Flynn

**Affiliations:** 1 Department of Pediatrics, Children’s Hospital of Pittsburgh of University of Pittsburgh Medical Center, Pittsburgh, Pennsylvania, United States of America; 2 Department of Microbiology and Molecular Genetics, University of Pittsburgh School of Medicine, Pittsburgh, Pennsylvania, United States of America; 3 Division of Laboratory Animal Resources, University of Pittsburgh, Pittsburgh, Pennsylvania, United States of America; 4 Department of Infectious Diseases Immunology, Statens Serum Institute, Copenhagen, Denmark; 5 Department of Immunology and Infectious Diseases, Harvard School of Public Health, Boston, Massachusetts, United States of America; McGill University Health Centre, CANADA

## Abstract

*Mycobacterium tuberculosis* infection presents across a spectrum in humans, from latent infection to active tuberculosis. Among those with latent tuberculosis, it is now recognized that there is also a spectrum of infection and this likely contributes to the variable risk of reactivation tuberculosis. Here, functional imaging with ^18^F-fluorodeoxygluose positron emission tomography and computed tomography (PET CT) of cynomolgus macaques with latent *M*. *tuberculosis* infection was used to characterize the features of reactivation after tumor necrosis factor (TNF) neutralization and determine which imaging characteristics before TNF neutralization distinguish reactivation risk. PET CT was performed on latently infected macaques (n = 26) before and during the course of TNF neutralization and a separate set of latently infected controls (n = 25). Reactivation occurred in 50% of the latently infected animals receiving TNF neutralizing antibody defined as development of at least one new granuloma in adjacent or distant locations including extrapulmonary sites. Increased lung inflammation measured by PET and the presence of extrapulmonary involvement before TNF neutralization predicted reactivation with 92% sensitivity and specificity. To define the biologic features associated with risk of reactivation, we used these PET CT parameters to identify latently infected animals at high risk for reactivation. High risk animals had higher cumulative lung bacterial burden and higher maximum lesional bacterial burdens, and more T cells producing IL-2, IL-10 and IL-17 in lung granulomas as compared to low risk macaques. In total, these data support that risk of reactivation is associated with lung inflammation and higher bacterial burden in macaques with latent Mtb infection.

## Author Summary

Asymptomatic infection with *Mycobacterium tuberculosis*, often called latent tuberculosis, affects more than 2 billion people. Reactivation of latent infection to active TB occurs in only a minority of those infected, yet can lead to deadly disease and transmission. Here we show, using a non-human primate model, that imaging using PET/CT can identify certain features that are associated with a higher risk of reactivation. These factors include overall lung inflammation, individual granulomas in the lung with higher bacterial burden, and a site of infection outside the lungs. Using these parameters may allow discovery of peripheral biomarkers regarding risk of reactivation from latent TB. Such biomarkers could identify those people who would benefit most from treatment of latent TB to prevent reactivation.

## Introduction

The vast majority of people infected with *Mycobacterium tuberculosis* (Mtb) develop asymptomatic, latent infection (LTBI). It is increasingly recognized that there is a spectrum of LTBI in humans, and this spectrum may correlate with the risk of reactivation [1]. Although reactivation risk is estimated at 10% per lifetime in HIV-negative LTBI humans, this is a population level estimate. Instead, it seems more likely that a small percentage of those with LTBI are at higher risk of reactivation. However, it has been challenging to identify the small fraction of the more than 2 billion latently infected humans who are at greatest risk of reactivation, so that therapy can be targeted to that population.

As in humans, LTBI in macaques is a stable, asymptomatic infection without clinical signs [2]. Reactivation of LTBI can be triggered in macaques by immune suppression due to SIV infection, TNF neutralization and CD4 depletion [3–6], but variable rates of reactivation are observed, similar to humans. We hypothesize that the spectrum of LTBI is associated with susceptibility to reactivation [1, 2]. Here we develop criteria based on ^18^F-fluorodeoxyglucose (FDG) positron emission tomography coupled with computed tomography (PET CT) imaging of macaques with LTBI to predict reactivation risk due to TNF neutralization. These criteria were then applied to latently infected macaques (without TNF neutralization) to identify biologic features that correlate with higher risk of reactivation. Macaques at high reactivation risk had greater cumulative lung bacterial burden, higher bacterial burden within an individual granuloma, more Mtb-infected mediastinal lymph nodes, and more T cells producing IL-2, IL-10 and IL-17 in lung granulomas compared to low risk macaques. Our results support the model of a spectrum of latency, suggesting that the extent and quality of bacterial control as well as lung inflammation in latency determines risk of reactivation after TNF neutralization.

## Results

### PET CT patterns of reactivation during TNF neutralization

**Fig 1:**
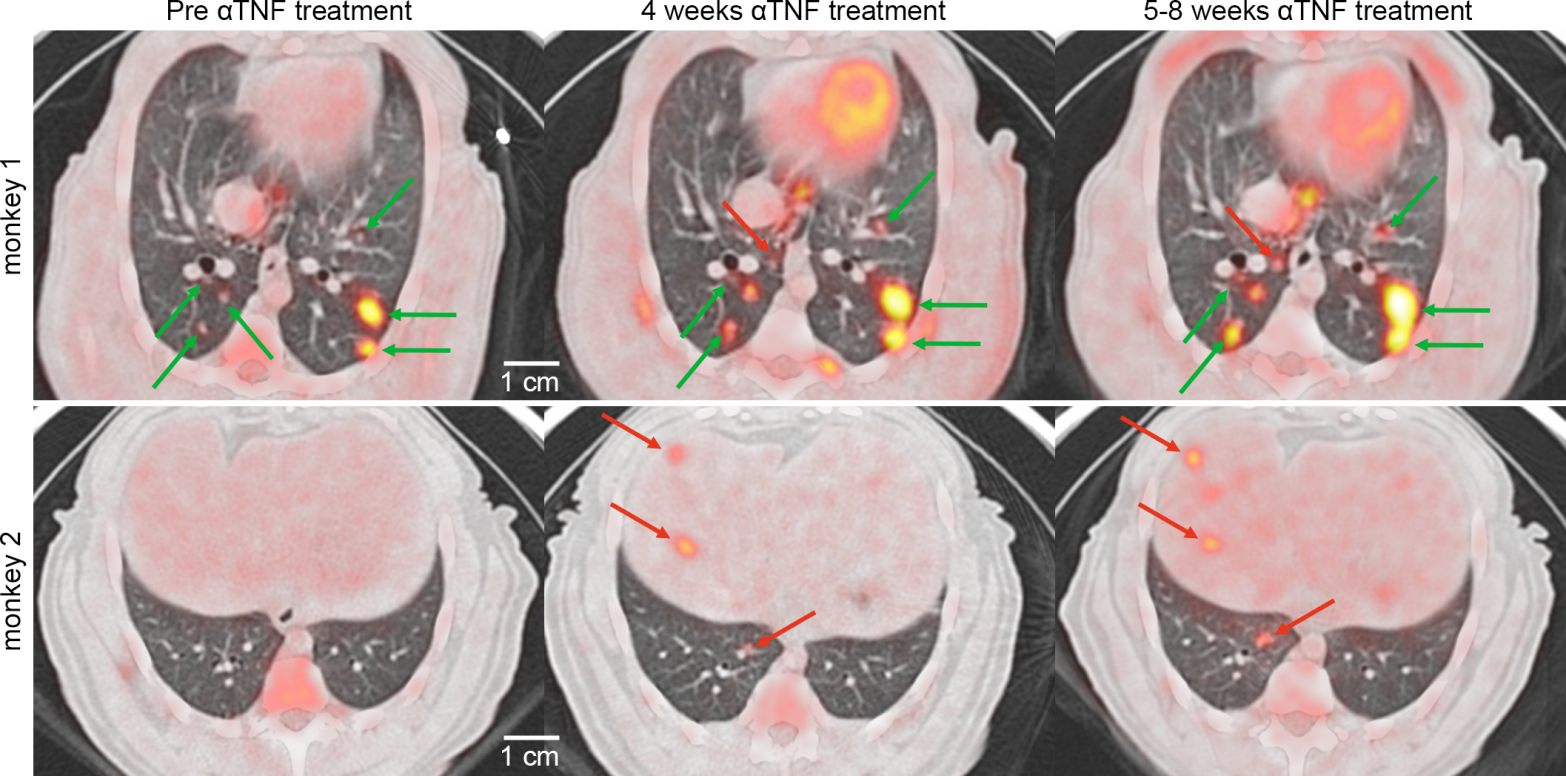
PET CT features of reactivation tuberculosis during TNF neutralization. Axial views of the lung are shown from two representative latently infected macaques with granulomas seen before (left panels, top and bottom) and during the course of TNF antibody treatment (middle and right panels, top and bottom). Pre-existing lesions (green arrows) within the same lung lobe can increase in size and FDG avidity or remain the same during the TNF neutralization. New lesions (red arrows) can arise in the lungs in an already involved lobe (top row) and/or in a new lobe or extrapulmonary sites such as the liver (red arrows, bottom row).

**Fig 2:**
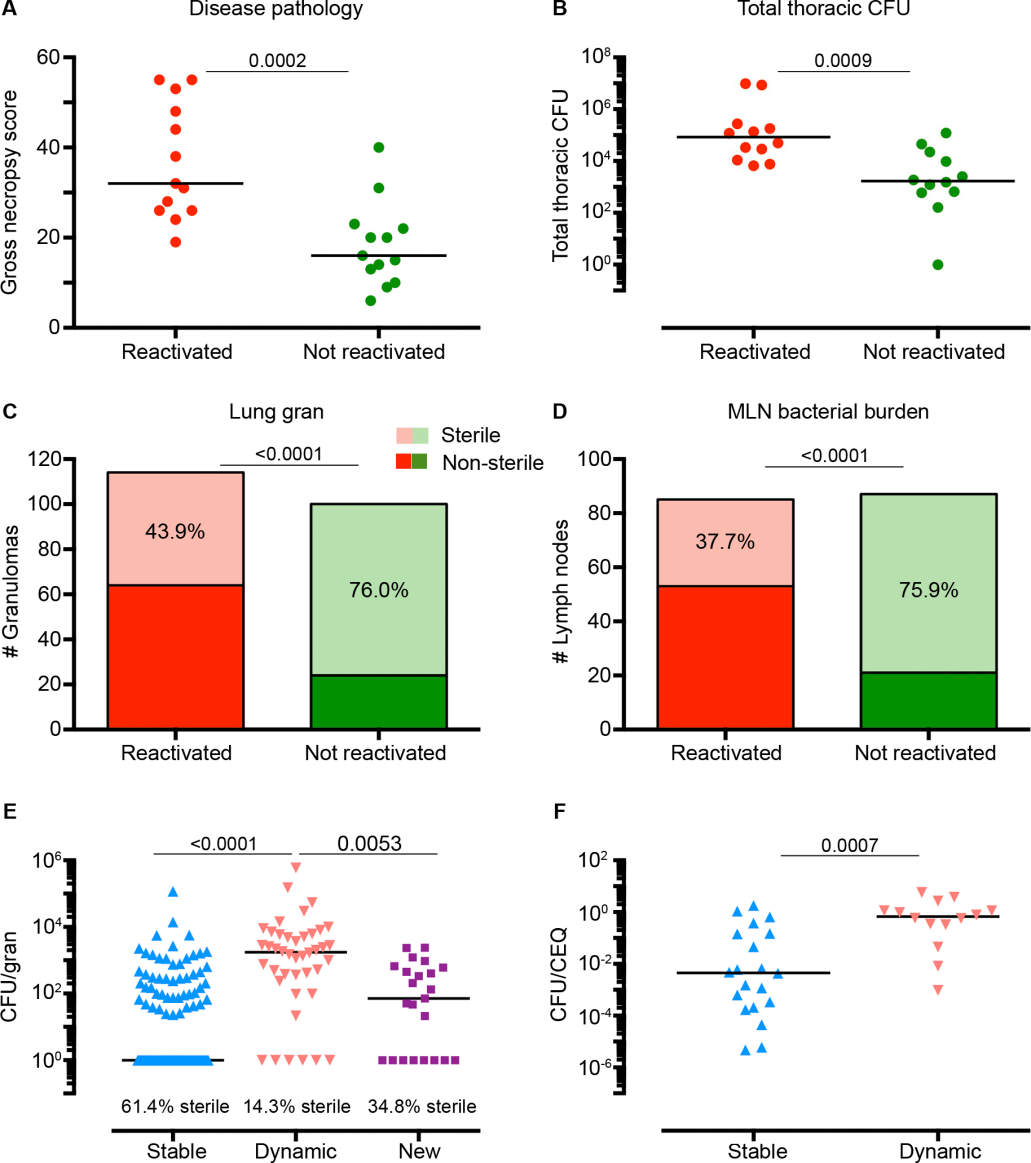
Disease pathology, bacterial burden and bacterial killing after TNF neutralization among reactivated and non-reactivated animals. (A) Macaques with radiographically defined reactivation have greater gross pathology at necropsy compared to animals that did not reactivate. Each symbol represents an animal. Statistics: Mann-Whitney (B) Thoracic bacterial burden (the sum of *Mtb* growth from all lung granulomas, other lung pathologies and all mediastinal lymph nodes, [MLN]) is higher in reactivated animals than in non-reactivated animals after TNF neutralization. Each symbol represents an animal. Statistics: Mann-Whitney (C) The number of granulomas among animals that developed reactivation (N = 13 macaques, 114 granulomas) are shown with the proportion of sterile granulomas reported in light green compared to animals that did not reactivate (N = 12 macaques, 100 granulomas). The p-value shown compares the proportion of sterile granulomas between groups of animals (Fisher’s Exact analysis). Up to 10 granulomas (randomized) are represented per animal to reduce bias. (D) Animals with reactivation had a smaller proportion of sterile MLNs compared to animals that did not reactivate. Proportions of sterile MLN are shown in the context of the total number of MLN in each group (reactivated group: N = 13 macaques, 85 MLNs vs non-reactivated group N = 13 macaques, 87 MLNs). The p-value shown compares the proportion of sterile granulomas between groups of animals (Fisher’s Exact analysis). Up tp 7 lymph nodes (randomized) are represented per animal to limit bias. (E) Dynamic granulomas (N = 42) had greater CFU per granuloma than stable (N = 132) and new (N = 23) granulomas after TNF neutralization. Percent sterile granulomas is noted for each group. Statistics: Kruskal-Wallis with Dunn’s multiple comparisons. (F) Dynamic lesions had less bacterial killing compared to stable lesions, as assessed by CFU/CEQ ratio. Statistics: Mann Whitney. The numbers indicated above each figure represents the p-value for the given statistical method.

We have previously published criteria for determining whether cynomolgus macaques with *M*. *tuberculosis* infection are “active” or “latent” by 6 months post-infection, based on clinical and microbiologic tests, as in humans [2, 7]. These clinical classifications were confirmed at necropsy, where those classified as active TB had significantly more pathology and bacterial burden than those classified as latent [2]. In this study, our aim was to determine whether we could identify latently infected macaques that would be more susceptible to reactivation. To do this, we employed serial FDG PET CT imaging, prior to and during neutralization of TNF, which we have shown previously can induce reactivation in macaques [5]. A cohort of cynomolgus macaques with LTBI (n = 26) was PET CT imaged at least 6 months post-infection, immediately prior to being randomly assigned to receive either TNF neutralizing antibody for 5–8 weeks or no treatment. Each macaque was evaluated for reactivation which was strictly defined here as dissemination, determined by the appearance of at least one new granuloma in lungs or extrapulmonary sites by PET CT during anti-TNF antibody treatment (Fig 1). Of 26 animals with TNF neutralization, 50% (n = 13) developed new lesions. At necropsy, macaques that developed new granulomas during TNF neutralization had greater disease pathology and higher total bacterial burden in lungs (Fig 2A and 2B) as well as within individual granulomas and lymph nodes (S1 Fig). Animals that developed reactivation had a significantly smaller proportion of sterile (or greater proportion with Mtb growth) among granulomas and mediastinal lymph nodes compared to animals that did not reactivate (Fig 2C and 2D). Thus, these data support the use of dissemination, the formation of new lesions in lungs or extrapulmonary sites, as a primary metric of reactivation.

While we defined reactivation in terms of bacterial dissemination, we postulated that we might also see evidence of loss of bacterial control among pre-existing lesions from the animals that reactivated. We examined lesion specific changes in metabolic activity (FDG avidity of each granuloma by PET, reported as standard uptake value, SUV) and/or size (by CT) during TNF neutralization. Granulomas were classified as “stable” if they remained similar in SUV (change < 5 units) or size (change < 1mm) or “dynamic” if they increased in SUV (≥ 5 units) or size (≥1mm) (S2 Fig). At least 1 dynamic lesion was observed during TNF neutralization in 69% (9 of 13) of reactivated monkeys compared to only 31% (4 of 13) among non-reactivated animals. Dynamic lesions were less likely to be sterile and had significantly higher bacterial burdens (measured as colony forming units, CFU) compared to stable lesions (Fig 2E) among all TNF-neutralized animals. The frequency of sterile lesions among new, stable and dynamic lesions was statistically different (Fig 2E) with the lower proportion of sterile lesions among the dynamic and newly developed granulomas. The total number of granulomas per monkey among reactivated (median = 12, IQR_25_ = 8,IQR_75_ = 24.5) and non-reactivated (median = 8, IQR_25_ = 4.5, IQR_75_ = 19.5) animals was similar (Mann-Whitney, p = 0.3). These data suggest that the increased bacterial burden observed in reactivation is not solely driven by the number of new lung granulomas but likely a combination of granuloma types and MLN burden.

We also compared the ratio of live Mtb CFU to chromosomal equivalents (CEQ) (the cumulative burden of live and dead Mtb) to estimate bacterial killing [8] in dynamic and stable lesions. Dynamic granulomas had higher CFU/CEQ ratios (i.e., less bacterial killing) than stable granulomas among all animals undergoing TNF neutralization (Fig 2F). Granulomas from reactivated animals had higher CFU/CEQ ratios compared to non-reactivated animals and LTBI controls (that did not receive TNF antibody) (S3 Fig). Importantly, however, many lesions in reactivated animals did not increase in metabolic activity or size or display reduced killing after TNF neutralization. This supports our previous data that demonstrates marked heterogeneity of lesions within an individual animal [8–11].

### PET CT patterns are associated with risk of reactivation of latent infection

**Fig 3:**
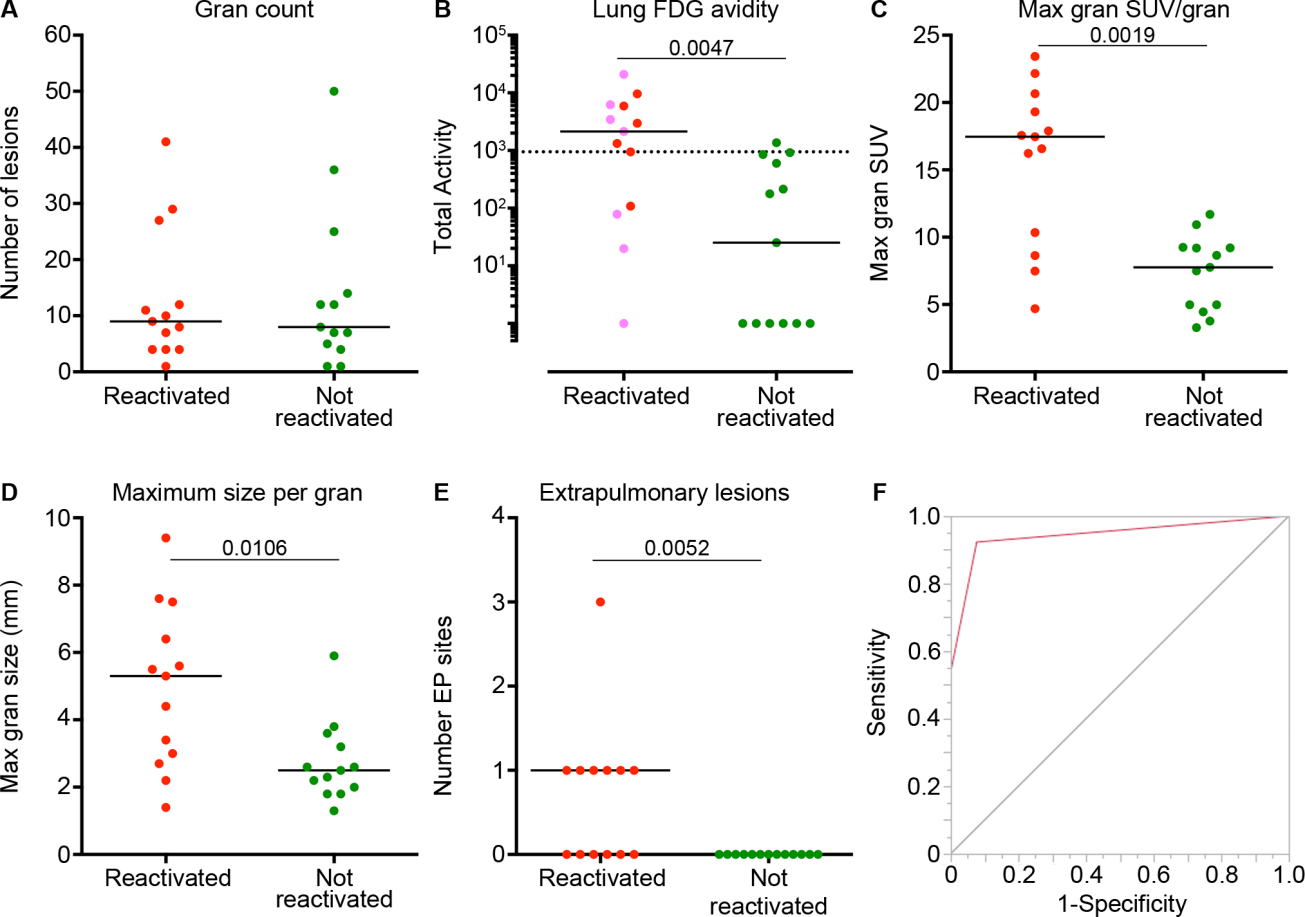
PET CT characteristics that distinguish reactivated and non-reactivated animals prior to TNF neutralization. (A) Similar numbers of lung granulomas are observed by PET CT in animals that reactivated and those that did not. (B) Total lung FDG avidity is higher prior to TNF neutralization in macaques that reactivated compared to those that did not. Macaques with an extrapulmonary lesion site (before TNF neutralization) are depicted are in pink. (C, D) The maximum FDG avidity (SUV) and size of any granuloma within a single monkey before TNF neutralization was greater among reactivator compared to non-reactivator animals. Each symbol represents an animal. (E) The number of extrapulmonary (EP) lesion sites before TNF neutralization was greater in animals that reactivated compared to those that did not. (F) A receiver operator curve (ROC) was calculated (AUC = 0.94) based on the presence of EP lesions and total lung FDG avidity (cut-off of 947.2 SUV) showing high sensitivity (92%) and specificity (92%). Panels A-D: each symbol represents a macaque. Statistics for A-E: Mann-Whitney. The numbers in each figure represents the p-value each analysis.

We next sought to identify PET CT characteristics of macaques prior to TNF neutralization that are predictive of reactivation risk. The number of lung granulomas observed before TNF neutralization was similar between animals that reactivated and those that did not (Fig 3A). We have previously shown that overall lung inflammation (total lung FDG avidity) detected by PET is loosely associated with lung bacterial burden in macaques with active TB, and decreased dramatically with anti-TB drug treatment [10, 12]. In this study, total lung FDG avidity immediately prior to anti-TNF treatment was significantly higher in animals that would later reactivate (Fig 3B) compared to those that did not. We then sought to define the distinguishing characteristics of individual lung granulomas prior to anti-TNF treatment that correlated with reactivation risk. Animals that would develop reactivation had a higher proportion of FDG avid (defined as SUV ≥ 5) granulomas (67.2%) compared to non-reactivator animals (30.8%) (Fisher’s Exact, p<0.0001) before TNF neutralization. Comparing the single most FDG avid or the largest size granuloma in each animal prior to anti-TNF treatment showed that those that would develop reactivation had a granuloma that was larger or had higher FDG avidity (Fig 3C and 3D). Although dynamic lesions had higher FDG avidity (median SUV 8.8, IQR _25–75_: 5.0, 13.9) compared to stable lesions (median SUV 4.5, IQR_25-75_: 2.9, 7.5, Mann-Whitney, p<0.0001) before TNF neutralization, we were unable to predict which lesions would become dynamic as only 23.7% of the lesions with greatest SUV and 31.6% of lesions of maximum size were dynamic lesions after TNF neutralization.

FDG avid mediastinal lymph nodes (MLN) seen on PET CT are usually associated with Mtb involvement. Previous data from our lab and from human studies suggest that infected MLN are a potential source of reactivation [5, 6, 13]. The cumulative FDG avidity of hot MLNs was significantly higher among animals that would later reactivate (reactivators cumulative SUV median = 9.6, IQR_25-75_: 5.8, 31.5 vs. non-reactivators cumulative SUV median = 5.5, IQR_25-75_: 0, 13.9, Mann-Whitney, p = 0.04). Lastly, the number of extrapulmonary sites of infection before TNF neutralization was significantly higher in animals that would later develop reactivation compared to those that would not (Fig 3E).

To assess which PET CT variables could distinguish reactivators from non-reactivators before TNF neutralization, we ran several different simple logistic regression models (and a contingency analysis) to narrow down the best predictors using the following variables: total lung FDG activity, number of “hot” lymph nodes, total number of lymph nodes, total SUV of lymph nodes, number of lesions, and the presence of extrapulmonary lesions. We chose total lung FDG activity and the presence of extrapulmonary lesions as best predictors based on goodness of fit. We then used recursive partitioning, splitting the data into a decision tree to define an optimal cut-off (947.2 SUV) for total lung FDG activity. Combining the presence of extrapulmonary involvement and total lung FDG avidity resulted in favorable receiver operator curve results (area under the curve = 0.94) and a high sensitivity (92.3%) and specificity (92.3%) (Fig 3F).

### Bacterial- and immunologic-associated risks of reactivation

**Fig 4:**
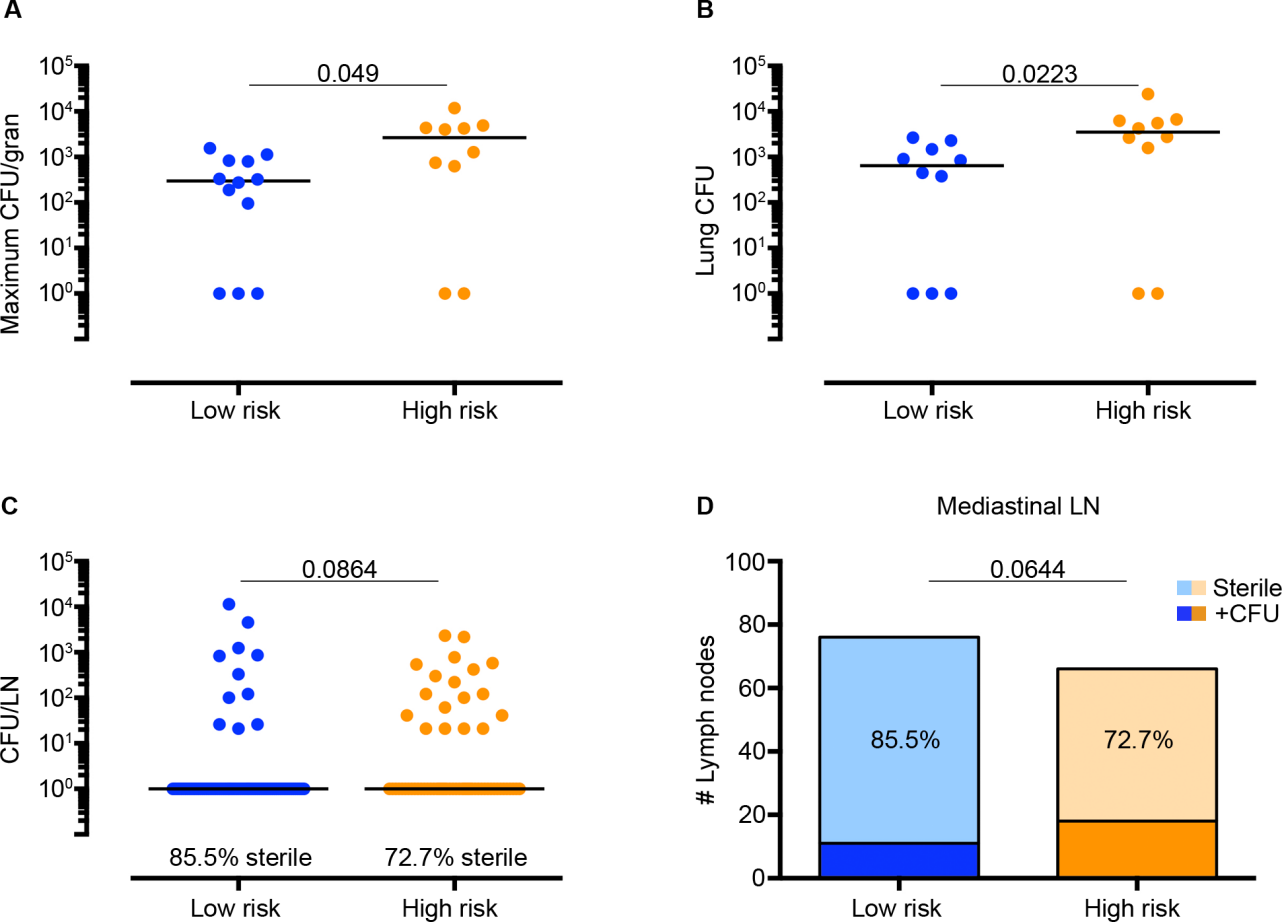
Parameters of reactivation risk in latently infected control macaques (without TNF neutralization). Animals were stratified for high or low risk of reactivation based on cut-offs determined by recursive partitioning (high = 947.2 Total FDG Activity or the presence of extrapulmonary lesions). (A) The maximum CFU per granuloma for an individual animal is greater in high-risk (N = 10) compared to low risk (N = 12) animals. (B) High risk LTBI control animals had higher total lung bacterial burden compared to low risk LTBI animals. (C) A trend toward higher CFU per individual MLN was observed in high-risk compared to low-risk LTBI animals. Up to 7 MLN are represented per animal to limit bias. (D) The proportion of sterile MLN observed among low and high risk LTBI controls is shown in the context of the total number of MLN per group. A greater proportion of sterile MLN was observed in low risk LTBI controls compared to high risk animals. The p-value shown compares the proportion of sterile MLN between groups of animals (Fisher’s Exact analysis). Statistics for A-C: Mann-Whitney.

It was not feasible to administer TNF neutralizing antibody to another large set of latently infected macaques to validate our predictions or identify biologic features associated with reactivation risk. Therefore we leveraged a set of latent control macaques (N = 25) that were necropsied concurrently with the TNF neutralized group, categorizing them being as at high or low risk for reactivation based on our PET CT defined parameters (i.e., total lung FDG avidity and extrapulmonary involvement) using the scan prior to necropsy. Animals who had evidence of extrapulmonary involvement on scan or a total lung FDG avidity of greater than or equal to 947.2 were classified as high risk. We then analyzed lesions from these animals to investigate the bacterial and immunological factors associated with risk of reactivation. While the median CFU per granuloma was the same between high and low risk animals (S4 Fig), a wide range of CFU per granuloma was observed within each individual monkey (S5 Fig). Because low risk animals appeared to have a lower peak bacterial burden compared to high risk, we then compared the maximum CFU per single granuloma within an in individual monkey to limit the bias. Interestingly, the maximum CFU per single granuloma within an animal was also higher among high risk compared to low risk animals (Fig 4A, S6 Fig). The total lung CFU (cumulative CFU of all lesions in the lung) was also greater among high-risk LTBI animals (Fig 4B, S6B Fig). High-risk LTBI animals also had a trend toward greater CFU per MLN (Fig 4C), which was surprising given the large range of CFU per MLN on an individual animal level (S7 Fig). High risk animals also had a smaller proportion of sterile MLN (i.e., greater proportion of MLN with Mtb growth) compared to low risk animals (Fig 4D). Together these data indicate that individual lesional characteristics (i.e., high bacterial burden in one granuloma or lymph node) are associated with high risk of reactivation.

**Fig 5:**
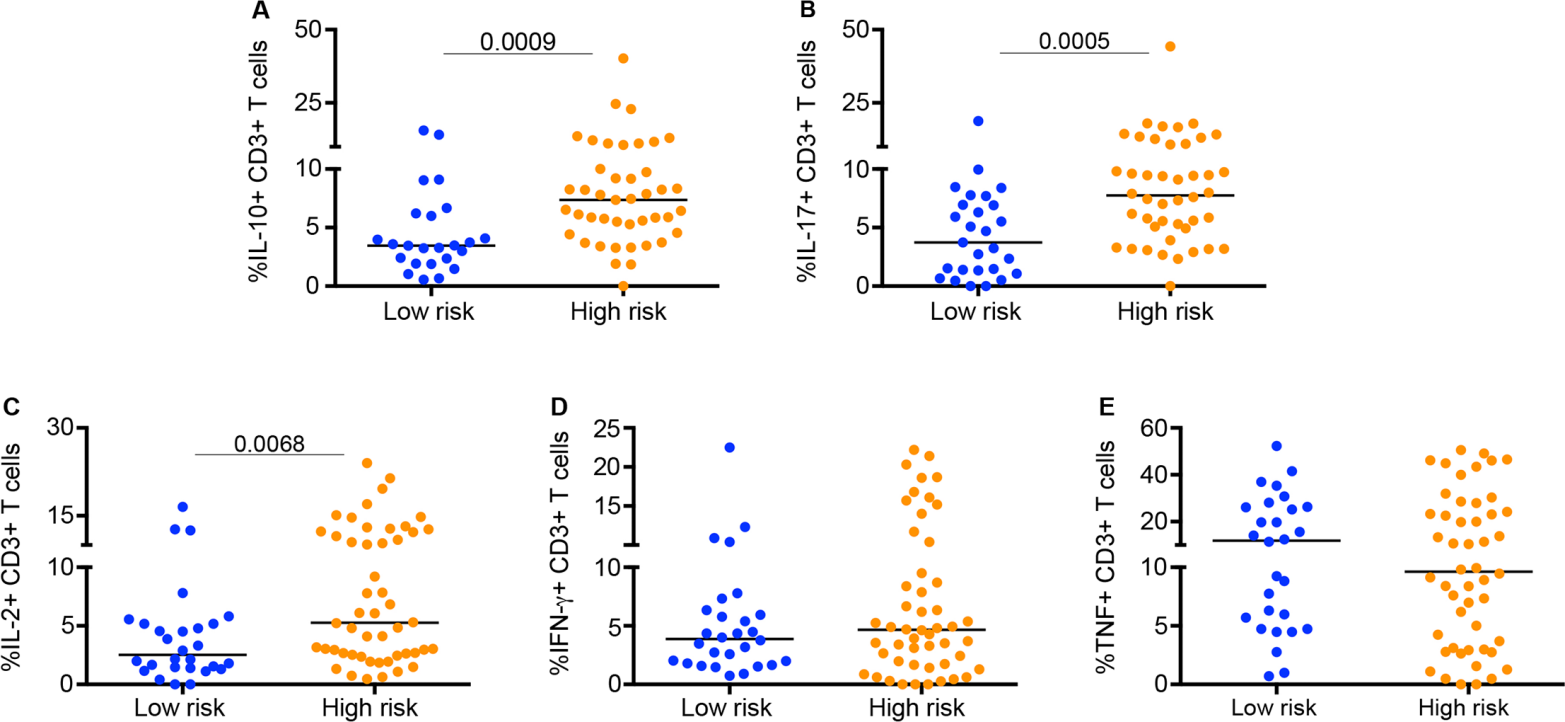
Frequencies of *Mycobacterium tuberculosis* (Mtb) specific T cells producing cytokines from individual granulomas of LTBI controls at high (N = 9) or low risk (N = 7) of reactivation. T cells from granulomas were stimulated with ESAT-6 and CFP-10 peptides. The frequency of T cells producing only IL-10 (A), only IL-17 (B) or only IL-2 (C) was greater in granulomas from high-risk compared to low risk LTBI animals. No differences were observed in IFN- γ (D) or TNF production (E). Each symbol represents a granuloma. Statistics: Mann-Whitney.

We then examined Mtb-specific T cell cytokine production within individual lung granulomas and blood of high- (n = 10) and low-risk (n = 10) LTBI control animals (without TNF neutralization). Granulomas from high-risk animals had higher frequencies of IL-17, IL-10 or IL-2 producing CD3+ T cells as compared to granulomas from low risk animals (Fig 5, S9 Fig). While most T cells from granulomas were single cytokine producers, as we previously described [9], we found that high-risk animals had a higher percentage of granuloma T cells producing more than one cytokine. There were no differences in frequencies of Mtb specific cytokine production by CD4+ and CD8+ T cells or by memory subsets in peripheral blood (S9, S10 and S11 Figs). These data suggest that granulomas from high-risk LTBI animals are more immune stimulated, possibly due to higher bacterial activity, although the specific factors driving this are unknown.

## Discussion

In this study, we strictly defined reactivation following TNF neutralization based on dissemination (formation of at least one new granuloma) and validated that definition at necropsy, where reactivated macaques had higher bacterial burden than those that did not reactivate. Together the data in this study support the hypothesis that the spectrum of latency has implications for risk of reactivation. Here we provide evidence that lung inflammation and/or evidence of extrapulmonary involvement detected by PET CT is associated with reactivation risk following TNF neutralization in macaques. In addition, reactivation risk is correlated with at least one granuloma of larger size or higher inflammation (measured by PET).

This unique set of data provided the opportunity to investigate individual granulomas in macaques predicted to be at high or low risk of reactivation. Using the parameters we developed based on PET CT characteristics of LTBI macaques that did or did not reactivate following TNF neutralization, we predicted the risk of reactivation of 25 latent control macaques. Neutralizing TNF would change the bacterial and immunologic features in these animals, which would prevent investigation of these factors in risk of reactivation. Therefore, instead of testing our prediction by treating the animals with anti-TNF treatment, we compared several factors in our predicted high or low risk animals without further intervention. Animals predicted at high risk had higher total lung and lymph node bacterial burden. In addition, an individual granuloma in high risk animals had a high bacterial burden, suggesting that a single poorly contained granuloma can contribute to reactivation. T cell cytokine production in granulomas was higher in high risk compared to low risk macaques in the absence of TNF neutralization. This could be due to more bacterial replication and antigen production in high risk animals. Alternatively, the combination of cytokines in certain granulomas from high risk animals may provide a less stable host immune environment. Further studies of immune responses in granulomas from low or high risk animals is necessary to differentiate cause and effect of cytokine responses in predisposing animals to reactivation. The data from this study suggests that only one or a few granulomas need to fail in bacterial containment to lead to dissemination and reactivation.

Not all granulomas were equally affected by TNF neutralization, suggesting that reactivation and dissemination can occur from as few as one unstable granuloma. For example, TNF neutralization led to dynamic granulomas (increasing in size or FDG avidity), but this was restricted to a subset of granulomas in reactivating monkeys. This is consistent with the independent and dynamic nature of lung granulomas in this model [8–11]. However, given the lack of current technology for tracking individual bacilli, it is not possible to confirm that dynamic lesions, or those with higher bacterial burden, are the source of dissemination. A limitation of this study is that we are unable at this time to identify direct causes of increased bacterial burden or instability of lesions, which are both associated with reactivation.

The ability of mediastinal lymph nodes (MLN) to control infection during clinical latency also appears to contribute to risk of reactivation. In LTBI, a Ghon complex refers to the combination of a lung granuloma and an involved lymph node, suggesting lymph node involvement is common in humans. Studies from the pre-antibiotic era also demonstrate substantial lymph node involvement in humans soon after infection [14]. Similarly, mediastinal lymph nodes have been detected by PET CT in humans with Mtb infection [15–18]. Thus, the role of lymph nodes in susceptibility to reactivation is likely important [19] and should be targeted in the development of drugs to treat latent infection. We previously published an association between extent of CD4 depletion in MLN and reactivation in latently infected macaques treated with CD4-depleting antibody [6]. In this current study, the latent controls predicted to be at high risk of reactivation a greater proportion of MLNs with Mtb growth compared to those that were at low risk. However, in a previous study, macaques vaccinated with BCG plus the protein fusion H56 vaccine were protected against TNF-neutralization induced reactivation [20]. Examination of our data from that study shows that protection against reactivation was associated with significantly fewer Mtb positive MLN (S12 Fig). Thus, it is likely that the MLN play an important role in reactivation risk. Even less is known about the presence of extrapulmonary sites of infection during LTBI. While it occurs in humans [21, 22], the actual prevalence has not been well described. We speculate that the events that result in extrapulmonary infection being established are due to poor initial control and early dissemination, which is then associated with reactivation risk.

In summary, we have provided evidence that the spectrum of clinically defined LTBI, specifically that associated with inflammation detected by PET CT and the presence of extrapulmonary disease, is associated with reactivation risk. It is likely that this occurs in humans with LTBI and similar lung lesions have been described in LTBI humans by PET CT [16, 23–25]. Importantly, this is the first assay that can functionally distinguish those at high and low risk for reactivation induced by TNF neutralization. We recognize that using PET CT to stratify reactivation risk is not feasible in most human settings. However, the use of PET CT in this well characterized animal model provides an opportunity to identify potential biomarkers in blood, including transcriptional signatures, which may correlate with reactivation risk. Prioritizing treatment to those patients at increased risk of reactivation (especially those with HIV infection) is a more efficient strategy in our current efforts to eradicate TB, as most programs are unable to provide treatment to all LTBI patients.

## Materials and Methods

### Animals

Adult (≥ 4 years of age) cynomolgus macaques (*Macacca fasicularis*) (Valley Biosystems, West Sacramento, CA) were screened with standard tests for co-morbidities prior to infection with Mtb as previously published[26]. Animals were maintained in a Biosafety Level 3 facility for primates after *M*. *tuberculosis* infection.

Cynomolgus macaques were infected with low dose (~25 CFU) of *M*. *tuberculosis* (Erdman strain) via bronchoscopic instillation into a lower lung lobe and subsequent serial clinical, microbiologic and immunologic parameters were followed until outcome was determined as previously described [2, 7]. Once animals were classified as latent, a subset was randomized to receive TNF neutralizing agent Adalimumb (Humira, Abbott Labs, Abbott Park, IL) at 4 mg/kg/dose subcutaneously every 7–10 days [5]. In general, PET-CT scans were performed at baseline before TNF neutralization and every 2 weeks after treatment until 5–8 weeks. A pre-necropsy scan was performed on all animals to facilitate harvesting scan-identified lesions. Serial analysis of these lesions before and during treatment was performed (see below). At necropsy, animals were maximally bled and gross pathology was assessed using our previously published quantitative scoring system in which a number is given for the size, number and pattern of granulomas in each lung lobe, mediastinal lymph node and extrapulmonary sites (e.g., liver, spleen). Harvested lung granulomas are characterized, measured and processed into single cell suspension for bacterial burden and flow cytometry as previously reported [2, 9].

### Ethics statement

All animal protocols and procedures were approved by the University of Pittsburgh’s Institutional Animal Care and Use Committee (protocol assurance number A3187-01.) Our specific protocol approval numbers for this project are 0808244, 0906877, 1011342,1105870 and 11080037. The IACUC adheres to national guidelines established in the Animal Welfare Act (7 U.S.C. Sections 2131–2159) and the Guide for the Care and Use of Laboratory Animals (8^th^ Edition) as mandated by the U.S. Public Health Service Policy.

### PET CT imaging and analysis

At predetermined time points, animals were sedated, intubated and imaged by 2-deoxy-2-^18^F-D-deoxyglucose (FDG) PET (microPET Focus 220 preclinical PET scanner, Siemens Molecular Solutions, Knoxville, TN) and CT (Neurologica Corp, Danvers, MA) imaging within our biosafety level 3 facility as previously described [10, 12, 27]. Lesions were identified by two analysts (M.T.C. and P.M.) and size was measured by CT. FDG avidity was measured by drawing a region of interest (ROI) in the axial view and SUVs (standard uptake volume, SUV = counts/(injected activity/body weight), normalized to muscle to reduce variability between scans, were calculated using OsiriX (Pixmeo, Geneva, Switzerland) as previously published [27].

The total lung FDG avidity was analyzed and calculated using Osirix viewer, an open-source PACS workstation and DICOM viewer. The whole lung was segmented on CT by using the Growing region algorithm on the OsiriX viewer to create a ROI of normal lung (Hounsfield units between -1024 and -200). The closing tool was used to include individual nodules and other pulmonary disease. The ROI was transferred to the co-registered PET scan and manually edited to ensure all pulmonary disease was included. All extrapulmonary structures and disease, including mediastinal lymph nodes, were excluded. Voxels outside the ROI were set to zero and voxels within the ROI with an SUV higher than normal lung (SUV ≥ 2.3) were isolated. These ROIs (capturing all SUV ≥ 2.3 within the lung) were exported into a spreadsheet using the OsiriX “Export ROI” plugin. Finally, the sum from the pixels of each slice (from the exported ROI) was calculated to represent the measurement of “Total Lung FDG Avidity”.

Granuloma specific changes observed before and during TNF neutralization were performed. “Stable” lesions were defined as lesions that maintained the same size and FDG avidity before and after TNF neutralization whereas “dynamic” lesions were those that that increased in size by at least 1mm or FDG avidity by at least 5 SUV. “New” lesions were not present baseline but appeared during the course of TNF neutralization.

### Isolation and quantification of *M*. *tuberculosis* genomes from tissue samples

Following necropsy, tissue sample homogenates were stored in PBS at -80°C. At processing, *M*. *tuberculosis* genomes were extracted with phenol-chloroform as previously described [8]. In brief, blinded samples were re-suspended in 1 mL Tris-EDTA buffer, pH 8.0, with 300 μl of 70°C UltraPure phenol:chloroform:isoamyl alcohol (25:24:1) (Invitrogen) and 250 μl of 0.1 mm zirconia-silica beads (BioSpec Products, Inc.). Tubes were mixed by inversion, incubated for 10 min, and twice vortexed for 4 min with a 1 min break at highest speed using a 24-tube vortex adaptor (MO BIO Laboratories, Inc.). Following a 10 min centrifugation at 14,000 RPM at 4°C, the aqueous layer of each sample was extracted and placed in a fresh tube with 50 μl of 5M sodium chloride. The phenol:chloroform:isoamyl alcohol extraction was repeated with 250 μl and a 30 min incubation at room temperature. After the incubation, samples were centrifuged once more at 14,000 RPM for 30 min at 4°C to separate off the aqueous phase. One volume of isopropanol and 1/10 volume of 3M sodium acetate (pH 5.2) was then added to each extraction to precipitate genomic DNA with an overnight incubation at -20°C. Each DNA pellet was washed with 70% ethanol and centrifuged for 30 min as before. Each pellet was then left to air-dry to remove excess ethanol and subsequently re-suspended in sterile nuclease free water (Ambion). DNA purity and concentration was measured using the Spectramax 190 spectrophotometer (Molecular Devices).

Quantification of chromosomal equivalents (CEQ) of *M*. *tuberculosis* was performed using real-time PCR of a single copy gene, Mtb *sigF*, with a previously described primer-probe combination [8]. The primer and probes for this target were purchased together in a pre-mixed PrimeTime qPCR assay (Integrated DNA Technologies). The sequences are as follows, 5’3’: probe–FAM-TCG GAC TTC GTC TCC TTC-Iowa Black, sigF Fwd–GCG GGT CGG GCT GGT CAA C, and sigF Rvs–CCT CGC CCA TGA TGG TAG GAA C. Real-time PCR was preformed in duplicate on the iQ5 Multicolor Real-Time PCR Detection System (Bio-Rad Laboratories, Inc.) and the 384well-capable 7900HT Fast Real Time PCR System (Applied Biosystems) with TaqMan Universal Master Mix II (Life Technologies). Precise determination of CEQ was derived from a standard curve of serially diluted *M*. *tuberculosis* genomic DNA prepared from liquid culture for each qPCR run. Real time PCR efficiency for each run was maintained between 90% and 110%.

While the quantification of both live Mtb and chromosomal equivalents are estimates, the ratios reflect a relative estimate of bacterial killing as published [8]. Accuracy of these estimates both in the detection of live bacteria and chromosomal equivalents may be limited by potential clumping of the bacteria resulting in underestimates of CFUs and minor variations in PCR amplification based on sample-specific differences in protein contamination or PCR inhibitors from blood.

### Flow cytometric analysis

Intracellular cytokine analyses were performed on individual granulomas harvested at necropsy and on PBMC at predetermine time points (6 months post infection). As previously described [9] single cell suspension of individual granulomas or PBMC were stimulated ex vivo with peptide pools of Mtb specific antigens ESAT-6 and CFP-10 (10 μg/ml of every peptide) or controls in the presence of Brefeldin A (Golgiplug: BD biosciences) for 3.5 hours (for granulomas) or 6 hours (for PBMCs) at 37°C with 5% CO2. For PBMC, Brefeldin A was added after 1 hour stimulation with Mtb antigens or controls. Positive control included stimulation with phorbol dibutyrate (PDBu) and ionomycin and negative controls included a media only control and an isotype controls (only for intracellular cytokine markers) for all PBMC samples and for granulomas whenever additional cells were available. For flow cytometry, cells from granulomas were initially stained for viability marker (Invitrogen) followed by cell surface marker CD3 (clone SP34-2; BD Pharmingen) for T cells. Cell surface markers for PBMC T cells included CD4 (clone L200, BD Horizon) and CD8 (clone SK1: BD biosciences) and markers for T cell memory subsets included CD45RA (clone 5H9, BD biosciences) and CD27 (clone O323, eBioscience). Intracellular cytokine staining panel for both granulomas and PBMC included Th1 pro-inflammatory cytokines: IFN-γ (clone B27), IL-2 (clone: MQ1-17H12), TNF (clone: MAB11); Th17 cytokine: IL-17 (clone eBio64CAP17) and regulatory cytokine: IL-10 (clone JES3-9D7) as previously described [9]. Data acquisition was performed using an LSR II (BD) and analyzed using FlowJo Software v.9.7 (Treestar Inc, Ashland, OR). For all PBMC T cells, the non-specific T cell response from the negative control (media) was subtracted from the Mtb specific antigen stimulated responses. MIATA guidelines were followed for sample collection and staining procedure for PBMC samples. The gating strategies used for the evaluation of granulomas and PBMC are described in S8 and S9 Figs. Intracellular cytokine data from granulomas in latent control animals (Fig 5) was previously published [9] but not analyzed based on risk of reactivation. PBMC T cell cytokine data (S10 and S11 Figs) from a subset of the animals was used for computational modeling and has been published [28].

### Bacterial burden determinations

Single cell suspension of each harvested site (i.e., lung granulomas, complex pathologies, grossly normal lung, MLN, liver, spleen) was plated on 7H11 plates (minimum detection level of *M*. *tuberculosis* burden was estimated at <10 Colony Forming Units per granuloma) as previously described [2, 8]. Specific bacterial burden of each site (granuloma, complex pathologies, or MLN) was calculated as the product of the bacterial burden on a per gram basis and the total mass of the tissue site.

Total thoracic burden was calculated as the sum of all *M*. *tuberculosis* growth from the lung (includes all individual granulomas and more complex pathologies such as consolidations, TB pneumonia, coalescing granulomas and clusters) and mediastinal lymph nodes. Total lung burden was calculated as the sum of all *M*. *tuberculosis* growth from lung lesions without mediastinal lymph nodes. The bacterial burden data were then transformed by adding 1 and reported as CFU.

### Statistical analysis

Normal distribution of the data was assessed for each continuous variable using the D’Agostino-Pearson Omnibus Test. For statistical comparison, pair-wise analysis of continuous data was performed by Student's T test for normally distributed data and Mann-Whitney test for nonparametric data. For analyses in which more than two groups were compared, Kruskall-Wallis test was performed with Dunn’s multiple comparisons as a post-hoc test of non-normally distributed data. Pair-wise analysis for matched data was performed using the Wilcoxon rank-sum test. P-values below 0.10 were reported specifically in figures and text.

To assess which PET CT variables could distinguish reactivators from non-reactivators before TNF neutralization, we ran several different simple logistic regression models (and a contingency analysis) to narrow down the best predictors using the following variables: total lung FDG activity, number of “hot” lymph nodes, total number of lymph nodes, total SUV of lymph nodes, number of lesions, and the presence of extrapulmonary lesions. We chose total lung FDG activity and the presence of extrapulmonary lesions as best predictors based on goodness of fit and then used recursive partitioning (a decision tree) to evaluate a cut-off for total lung FDG activity. A receiver operating characteristic (ROC) curve was plotted in order to graphically represent the sensitivity and specificity of the combination of these two predictors. Statistical analysis was performed using Prism 6.0 (Graphpad Software, Inc). The ROC curve was plotted in JMP Pro 10.2 (SAS Institute Inc.).

Given the variability in the number of granulomas per animals, methods were developed to minimize the potential for bias among animals that had a greater number of lesions (i.e., granulomas and MLN) for analysis. We derived a number of representative samples per monkey by first calculating the median number of samples (for which we had bacterial burden) per monkey per group so that one monkey could not contribute more than the median number of samples per monkey.

## Supporting Information

S1 Fig (A) Granuloma colony forming units (CFU) are higher in reactivated (N = 13 macaques, 114 granulomas) compared to non-reactivated (N = 12 macaques, 100 granulomas) animals. Each symbol represents an individual granuloma. The number of sterile granulomas is noted for each group. 10 granulomas (randomized) are represented per animal. (B) CFU of individual MLN are greater in reactivated (N = 13 macaques, 85 MLNs) compared to non-reactivated (N = 13 macaques, 87 MLNs) animals. Each symbol represents an individual MLN and 7 lymph nodes (randomized) are represented per animal. Percent sterile MLN is noted for each group. The p-value indicated above reflects a comparison of the median CFU per granuloma or MLN between groups (Mann-Whitney).
(TIF)

S2 Fig Radiographically dynamic and stable lesions of latently infected macaques during neutralization. PET CT images from a macaque before and during TNF neutralization show examples of “stable” lung granulomas (top row, yellow arrows) defined has having no substantial change in FDG avidity or size. In contrast, in a different lung location in this same animal, dynamic lung lesions are seen (bottom row, yellow arrows) and are defined as granulomas that increase substantially in size (≥1mm) and/or FDG avidity (≥ 5 units) during the course of TNF neutralization.
(TIF)

S3 Fig Bacterial killing in granulomas is reduced in reactivated macaques compared to those that did not reactivate following TNF neutralization or LTBI controls. The ratio of live *M*. *tuberculosis* colony forming units (CFU) to chromosomal equivalents (genomic quantities of both live and dead *M*. *tuberculosis*) is used to estimate bacterial growth and killing. Less killing (or higher CFU/CEQ ratio) is observed among granulomas from reactivated animals compared to non-reactivated animals given TNF antibody as well as from latently infected control animals (LTBI) not given anti-TNF antibody. P-values shown were performed by Kruskall-Wallis with post-hoc Dunn’s multiple comparison. Open symbols represent dynamic lesions, closed symbols represent stable lesions, and pink shaded circles represent new lesions.
(TIF)

S4 Fig Bacterial burden per granuloma is higher in high risk compared to low risk macaques without anti-TNF treatment. Animals were stratified for high (N = 10) and low (N = 12) risk of reactivation. (A) Bacterial burden per granulomas (CFU/gran) was greater in high risk compared to low risk LTBI animals although the medians were the same (median = 1). 10 granulomas (randomized) are represented per animal. The proportion of sterile granulomas is noted for each group. The p-value shown was performed by Mann-Whitney. (B) The proportion of sterile granulomas is shown relative to the total number of granulomas from all monkeys in each risk group. A trend toward a lower percentage of sterile granulomas was observed in high risk animals compared to low risk. The p-value reflects the proportion of sterile granulomas in each risk group (Fisher’s Exact).
(TIF)

S5 Fig Range of bacterial burden per granuloma (CFU/gran) for each high (N = 10) and low (N = 12) risk latent control macaque. Each symbol represents an individual granuloma. Numbers along the x-axis represent individual animal identifiers.
(TIF)

S6 Fig Linear scale of maximum CFU per granuloma and total lung bacterial burden. Animals were stratified for high or low risk of reactivation based on cut-offs determined by recursive partitioning (high = 947.2 Total FDG Activity or the presence of extrapulmonary lesions). (A) The maximum CFU per granuloma for an individual animal is greater in high-risk (N = 10) compared to low risk (N = 12 animals. (B) High risk LTBI control animals had higher total lung bacterial burden compared to low risk LTBI animals. The indicated p-values are derived by Mann-Whitney. Each symbol represents an animal. Data are the same as in Fig 4A and 4B but show a linear scale of bacterial burden.
(TIF)

S7 Fig Range of bacterial burden per mediastinal LN (CFU/LN) for each high (N = 10) and low (N = 12) risk latent control animal. Each symbol represents an individual MLN. Numbers along the x-axis represent individual animal identifiers.
(TIFF)

S8 Fig Gating strategy for surface and intracellular cytokine staining in lung granulomas. After granulomas are harvested at necropsy and homogenized into single cell suspension, viable cells were negatively selected based on the absence of viability marker (**A**). Lymphocytes were selected based on SSC and FSC (i.e., size and granularity) (**B**). CD3+ were gated on the lymphocyte population and defined as T cells (**C**) from which cytokine producing T cells (**D-F**) were gated as in the example shown. Arrow indicates sequence of gating.
(TIF)

S9 Fig Gating strategy for surface and intracellular cytokines staining of macaques PBMCs. After PMBC was purified by percoll density gradient, cells were stimulated with Mtb specific antigens or controls for 6 hours and stained for flow cytometry. From an arbitrary live cell gate (A), Lymphocytes (B) were selected based on SSC and FSC (i.e., size and granularity). CD4 or CD8 T cells (C) were gated on the lymphocyte population. From either CD4 or CD8 T cells memory subsets (D) were selected based on CD45RA and CD27 markers as follows: CD45RA+CD27+ as Naïve, CD45RA-CD27+ as Central memory, CD45RA-CD27- as Effector memory and CD45RA+CD27- as Effector or terminally differentiated. Cytokine producing CD4 or CD8 T cells or memory subsets were gated as shown (E-G) in the example shown here for ESAT-6+CFP-10 stimulated cells with specific cytokine marker labeled antibody or isotype. Arrows indicates sequence of gating.
(TIF)

S10 Fig Intracellular cytokine staining of peripheral blood CD4 and CD8 T cells among low (N = 8 macaques, blue) and high (N = 7 macaques, red) risk latently infected animals. Mycobacterial antigen (ESAT6 and CFP10) specific production of single cytokine staining of IFN-γ, IL-2, and TNF was determined from central memory (CD27+CD45Ra -), effector memory (CD27-CD45Ra-), and terminal effector (CD27-CD45Ra+) populations of CD4 and CD8 T cells. There were no differences in cytokine expression between high and low risk animals. No differences were observed between high and low risk animals.
(TIF)

S11 Fig Intracellular cytokine staining of peripheral blood CD4 and CD8 T cells among low (N = 8 macaques, blue) and high (N = 7 macaques, red) risk latently infected animals. Polyfunctional cytokine (IFN-γ, IL-2, and TNF) staining of mycobacterial antigen (ESAT6 and CFP10) specific production T cells was examined among central memory (CD27+CD45Ra -), effector memory (CD27-CD45Ra-), and terminal effector (CD27-CD45Ra+) populations of CD4 and CD8 T cells. No differences were observed between high and low risk animals.
(TIF)

S12 Fig Vaccine-induced protection from reactivation is associated with sterilized MLN. In a previously published vaccine trial [16], macaques were vaccinated with either BCG, a combination of BCG and H56 or were unvaccinated. Latent animals from each group were then treated with anti-TNF antibody; 3 of 4 BCG-vaccinated animals reactivated, similar to latent controls in that study while 0 of 4 BCG/H56 vaccinated animals reactivated. Here, the frequency of MLN with growth of *M*. *tuberculosis* was analyzed from those animals. The BCG/H56 animals had a significantly lower proportion of Mtb-positive MLN than the control animals. The indicated p-value is derived from Kruskal Wallis with Dunn’s multiple comparison.
(TIF)
