# Supplementary figures and images for: PET CT Identifies Reactivation Risk in Cynomolgus Macaques with Latent M. tuberculosis
Source: PLoS Pathog. 2016 Jul 5;12(7):e1005739. doi: 10.1371/journal.ppat.1005739 (PMC4933353; doi:10.1371/journal.ppat.1005739)

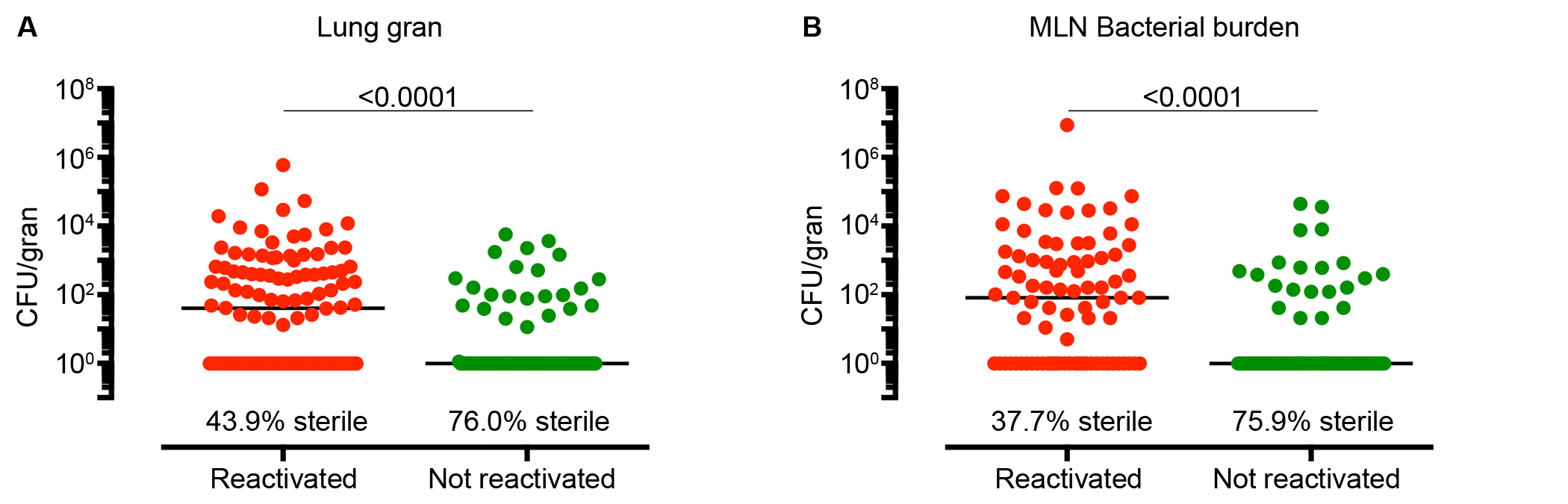

Supplement: S1 Fig — (A) Granuloma colony forming units (CFU) are higher in reactivated (N = 13 macaques, 114 granulomas) compared to non-reactivated (N = 12 macaques, 100 granulomas) animals. Each symbol represents an individual granuloma. The number of sterile granulomas is noted for each group. 10 granulomas (randomized) are represented per animal. (B) CFU of individual MLN are greater in reactivated (N = 13 macaques, 85 MLNs) compared to non-reactivated (N = 13 macaques, 87 MLNs) animals. Each symbol represents an individual MLN and 7 lymph nodes (randomized) are represented per animal. Percent sterile MLN is noted for each group. The p-value indicated above reflects a comparison of the median CFU per granuloma or MLN between groups (Mann-Whitney). (TIF) [file ppat.1005739.s001.tif]

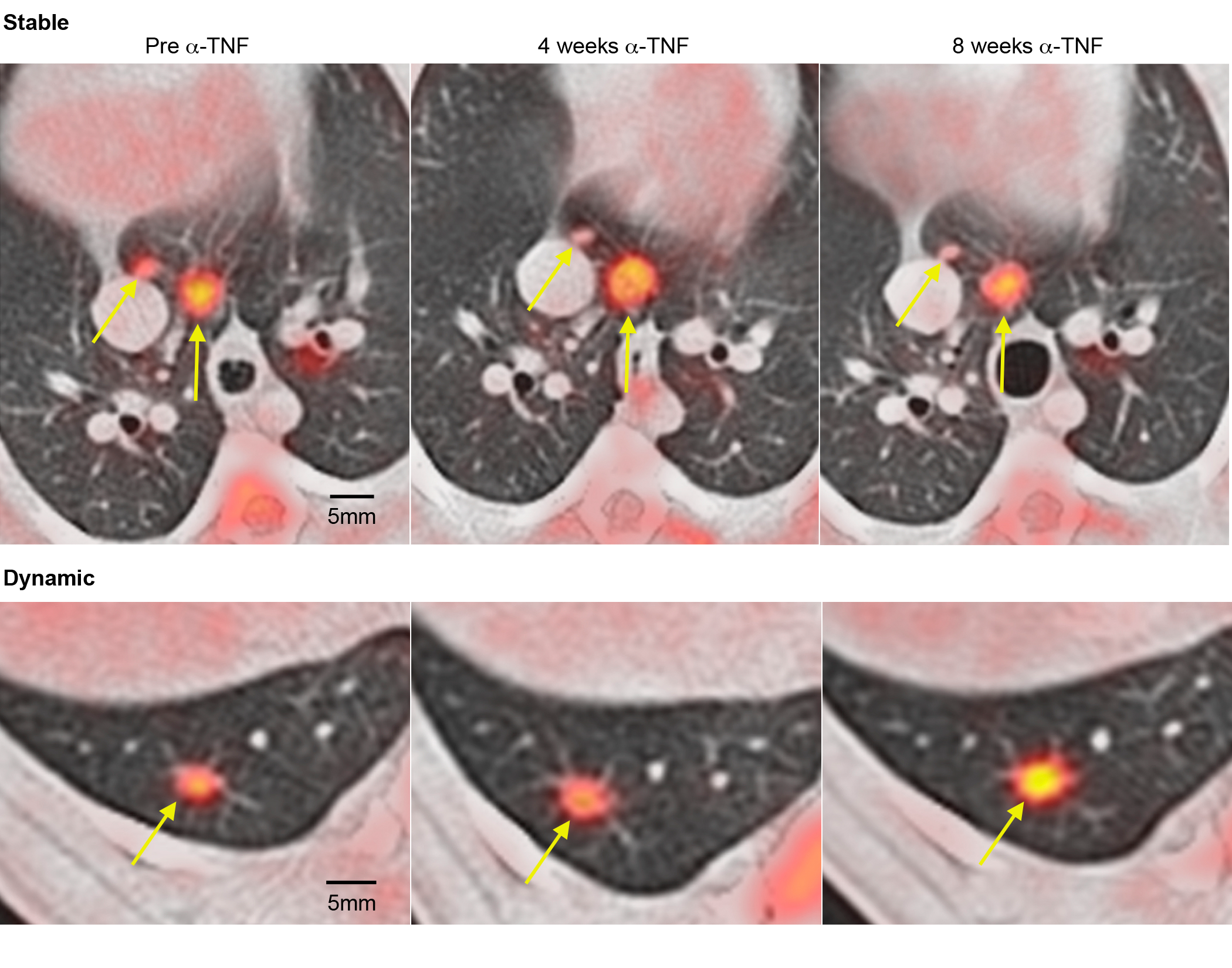

Supplement: S2 Fig — PET CT images from a macaque before and during TNF neutralization show examples of “stable” lung granulomas (top row, yellow arrows) defined has having no substantial change in FDG avidity or size. In contrast, in a different lung location in this same animal, dynamic lung lesions are seen (bottom row, yellow arrows) and are defined as granulomas that increase substantially in size (≥1mm) and/or FDG avidity (≥ 5 units) during the course of TNF neutralization. (TIF) [file ppat.1005739.s002.tif]

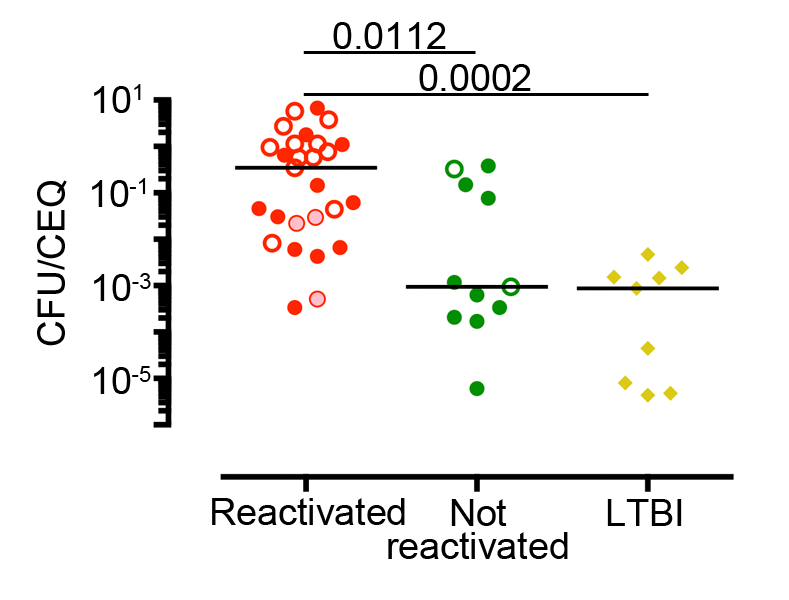

Supplement: S3 Fig — The ratio of live M. tuberculosis colony forming units (CFU) to chromosomal equivalents (genomic quantities of both live and dead M. tuberculosis) is used to estimate bacterial growth and killing. Less killing (or higher CFU/CEQ ratio) is observed among granulomas from reactivated animals compared to non-reactivated animals given TNF antibody as well as from latently infected control animals (LTBI) not given anti-TNF antibody. P-values shown were performed by Kruskall-Wallis with post-hoc Dunn’s multiple comparison. Open symbols represent dynamic lesions, closed symbols represent stable lesions, and pink shaded circles represent new lesions. (TIF) [file ppat.1005739.s003.tif]

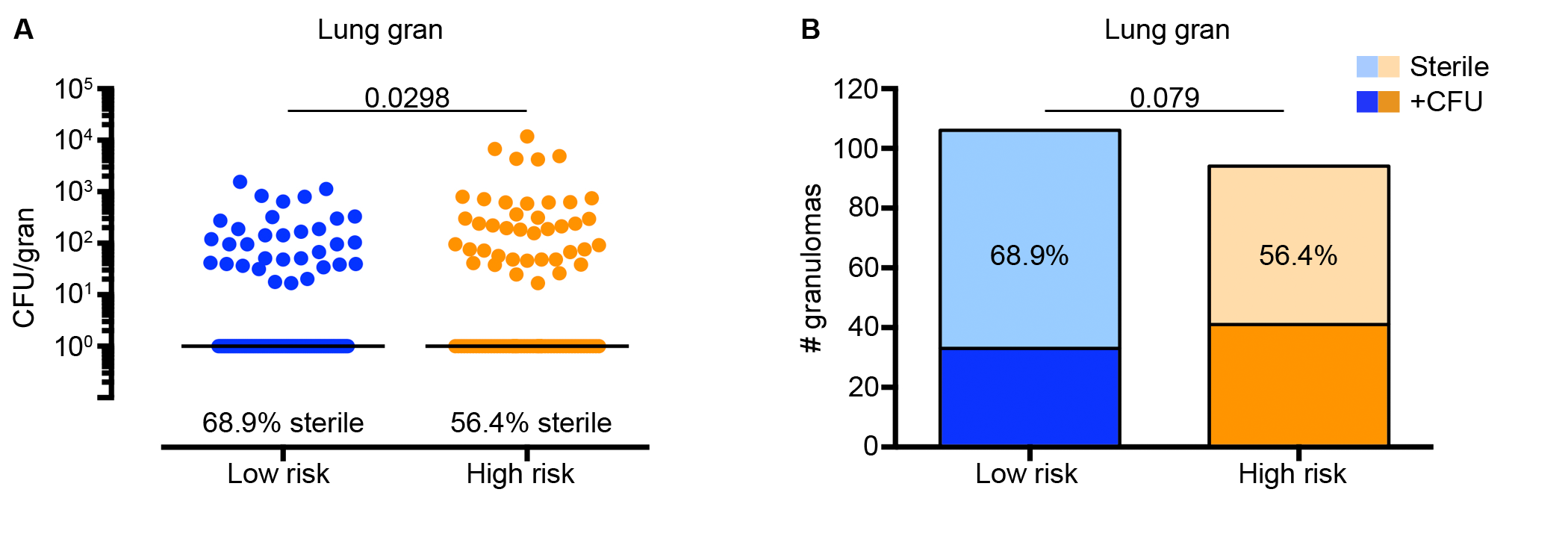

Supplement: S4 Fig — Animals were stratified for high (N = 10) and low (N = 12) risk of reactivation. (A) Bacterial burden per granulomas (CFU/gran) was greater in high risk compared to low risk LTBI animals although the medians were the same (median = 1). 10 granulomas (randomized) are represented per animal. The proportion of sterile granulomas is noted for each group. The p-value shown was performed by Mann-Whitney. (B) The proportion of sterile granulomas is shown relative to the total number of granulomas from all monkeys in each risk group. A trend toward a lower percentage of sterile granulomas was observed in high risk animals compared to low risk. The p-value reflects the proportion of sterile granulomas in each risk group (Fisher’s Exact). (TIF) [file ppat.1005739.s004.tif]

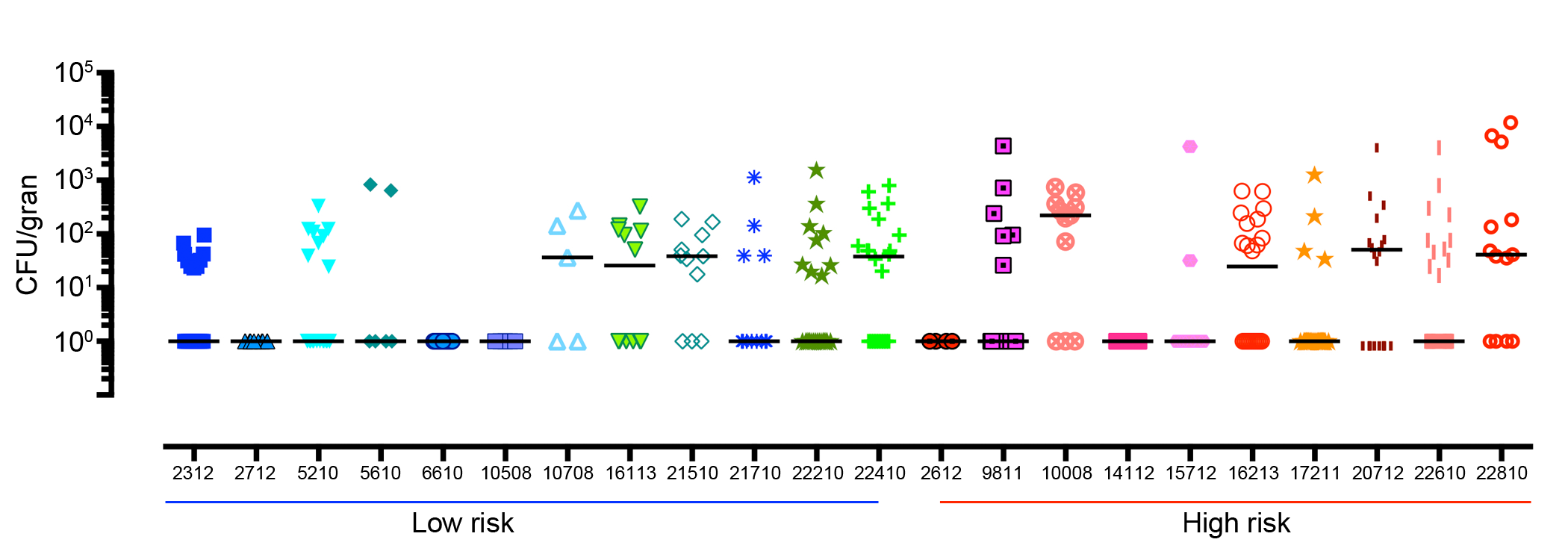

Supplement: S5 Fig — Each symbol represents an individual granuloma. Numbers along the x-axis represent individual animal identifiers. (TIF) [file ppat.1005739.s005.tif]

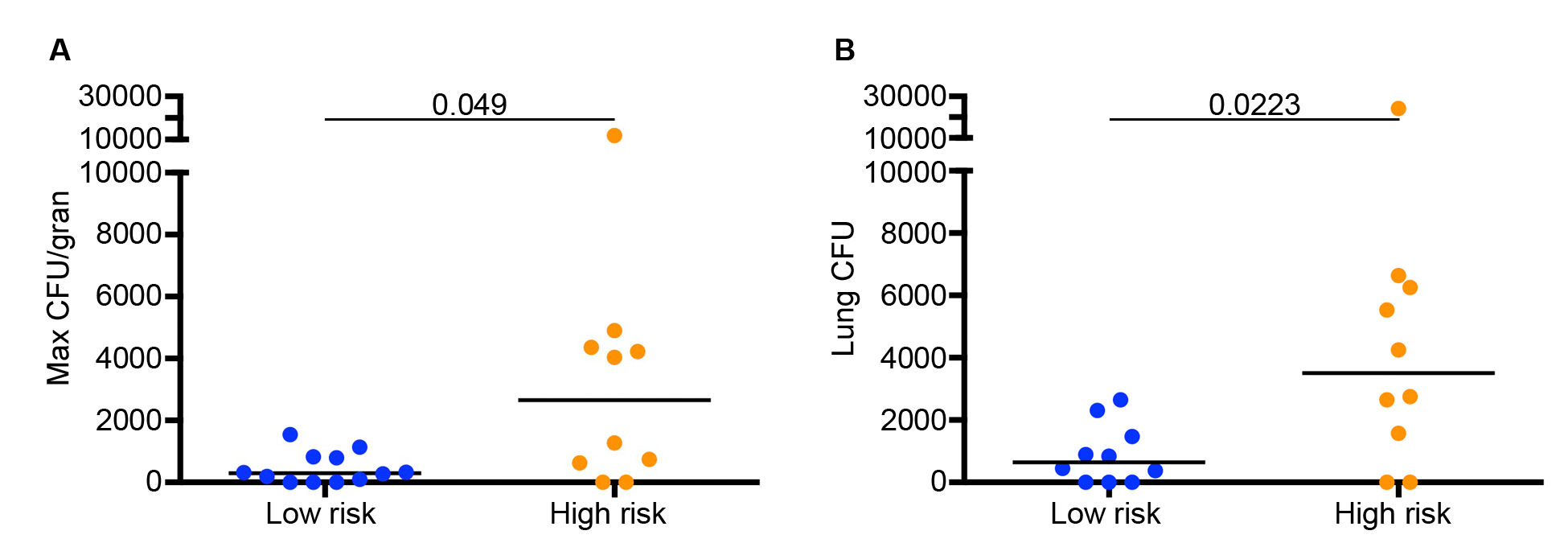

Supplement: S6 Fig — Animals were stratified for high or low risk of reactivation based on cut-offs determined by recursive partitioning (high = 947.2 Total FDG Activity or the presence of extrapulmonary lesions). (A) The maximum CFU per granuloma for an individual animal is greater in high-risk (N = 10) compared to low risk (N = 12 animals. (B) High risk LTBI control animals had higher total lung bacterial burden compared to low risk LTBI animals. The indicated p-values are derived by Mann-Whitney. Each symbol represents an animal. Data are the same as in Fig 4A and 4B but show a linear scale of bacterial burden. (TIF) [file ppat.1005739.s006.tif]

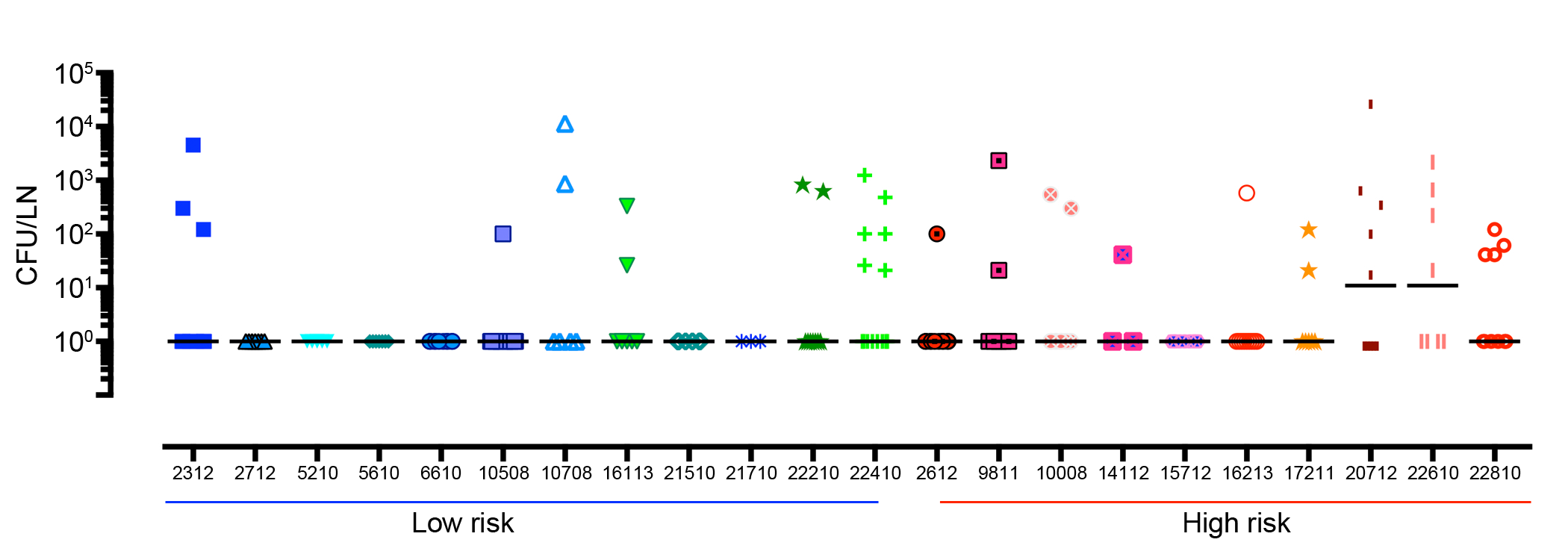

Supplement: S7 Fig — Each symbol represents an individual MLN. Numbers along the x-axis represent individual animal identifiers. (TIFF) [file ppat.1005739.s007.tiff]

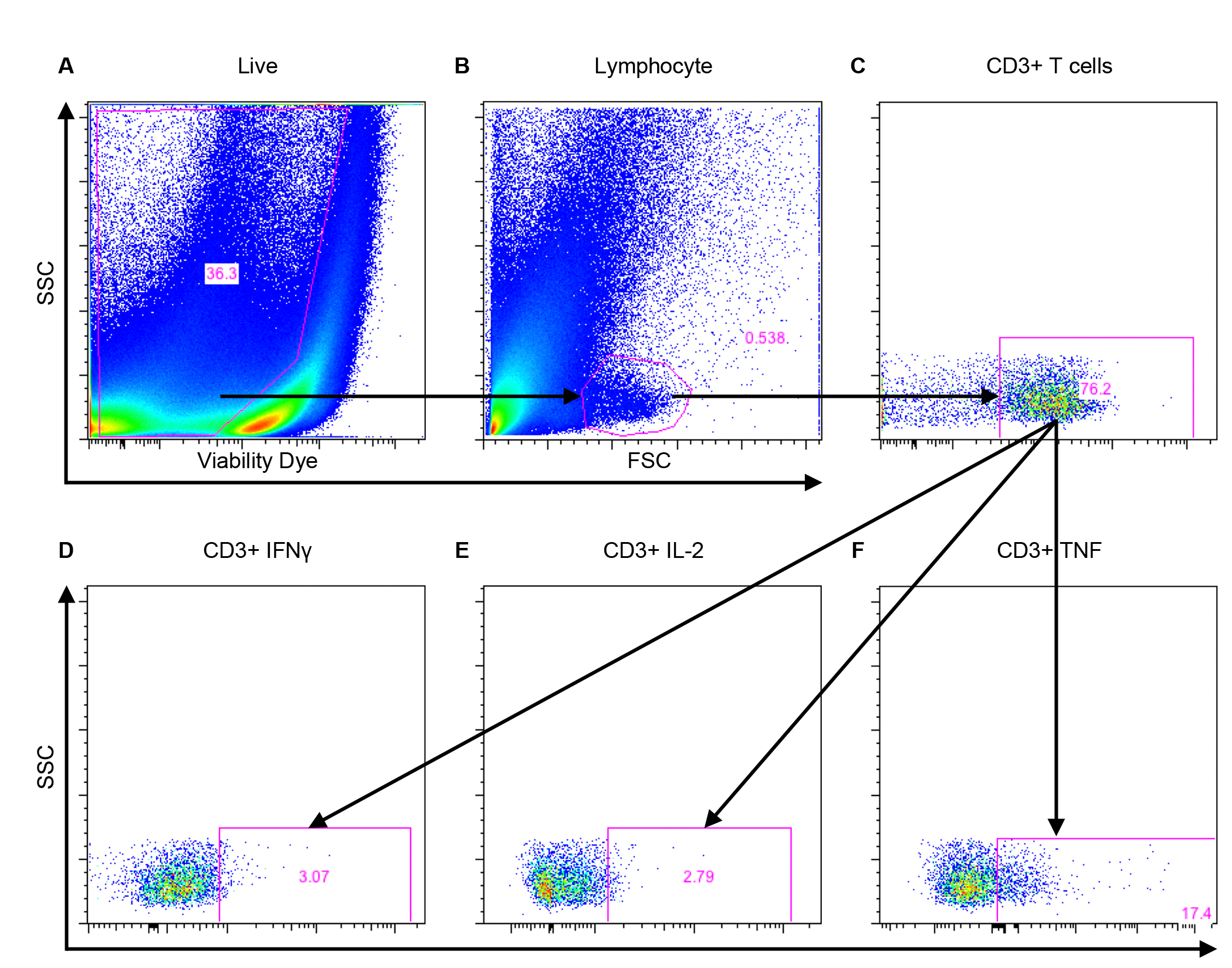

Supplement: S8 Fig — After granulomas are harvested at necropsy and homogenized into single cell suspension, viable cells were negatively selected based on the absence of viability marker (A). Lymphocytes were selected based on SSC and FSC (i.e., size and granularity) (B). CD3+ were gated on the lymphocyte population and defined as T cells (C) from which cytokine producing T cells (D-F) were gated as in the example shown. Arrow indicates sequence of gating. (TIF) [file ppat.1005739.s008.tif]

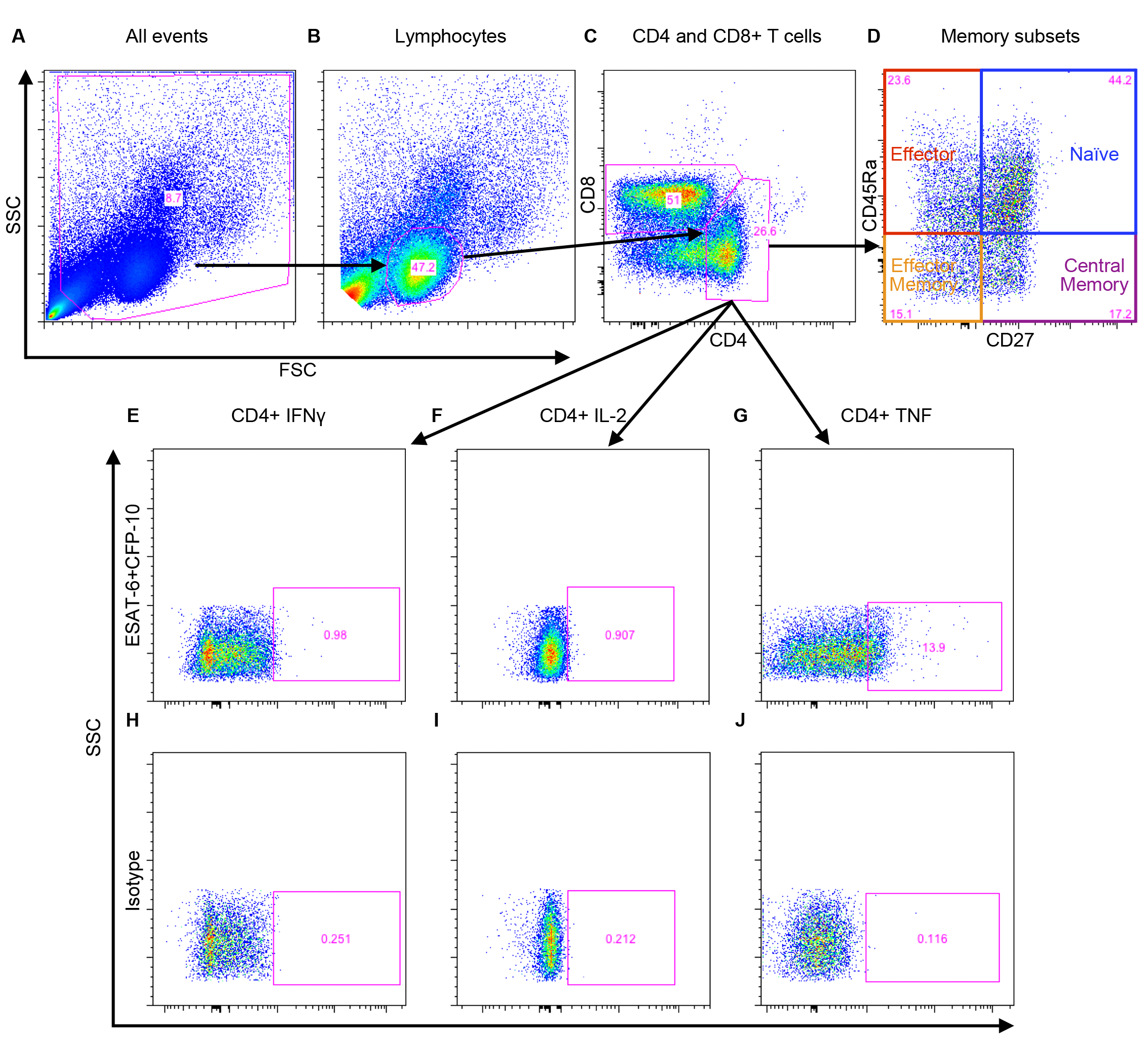

Supplement: S9 Fig — After PMBC was purified by percoll density gradient, cells were stimulated with Mtb specific antigens or controls for 6 hours and stained for flow cytometry. From an arbitrary live cell gate (A), Lymphocytes (B) were selected based on SSC and FSC (i.e., size and granularity). CD4 or CD8 T cells (C) were gated on the lymphocyte population. From either CD4 or CD8 T cells memory subsets (D) were selected based on CD45RA and CD27 markers as follows: CD45RA+CD27+ as Naïve, CD45RA-CD27+ as Central memory, CD45RA-CD27- as Effector memory and CD45RA+CD27- as Effector or terminally differentiated. Cytokine producing CD4 or CD8 T cells or memory subsets were gated as shown (E-G) in the example shown here for ESAT-6+CFP-10 stimulated cells with specific cytokine marker labeled antibody or isotype. Arrows indicates sequence of gating. (TIF) [file ppat.1005739.s009.tif]

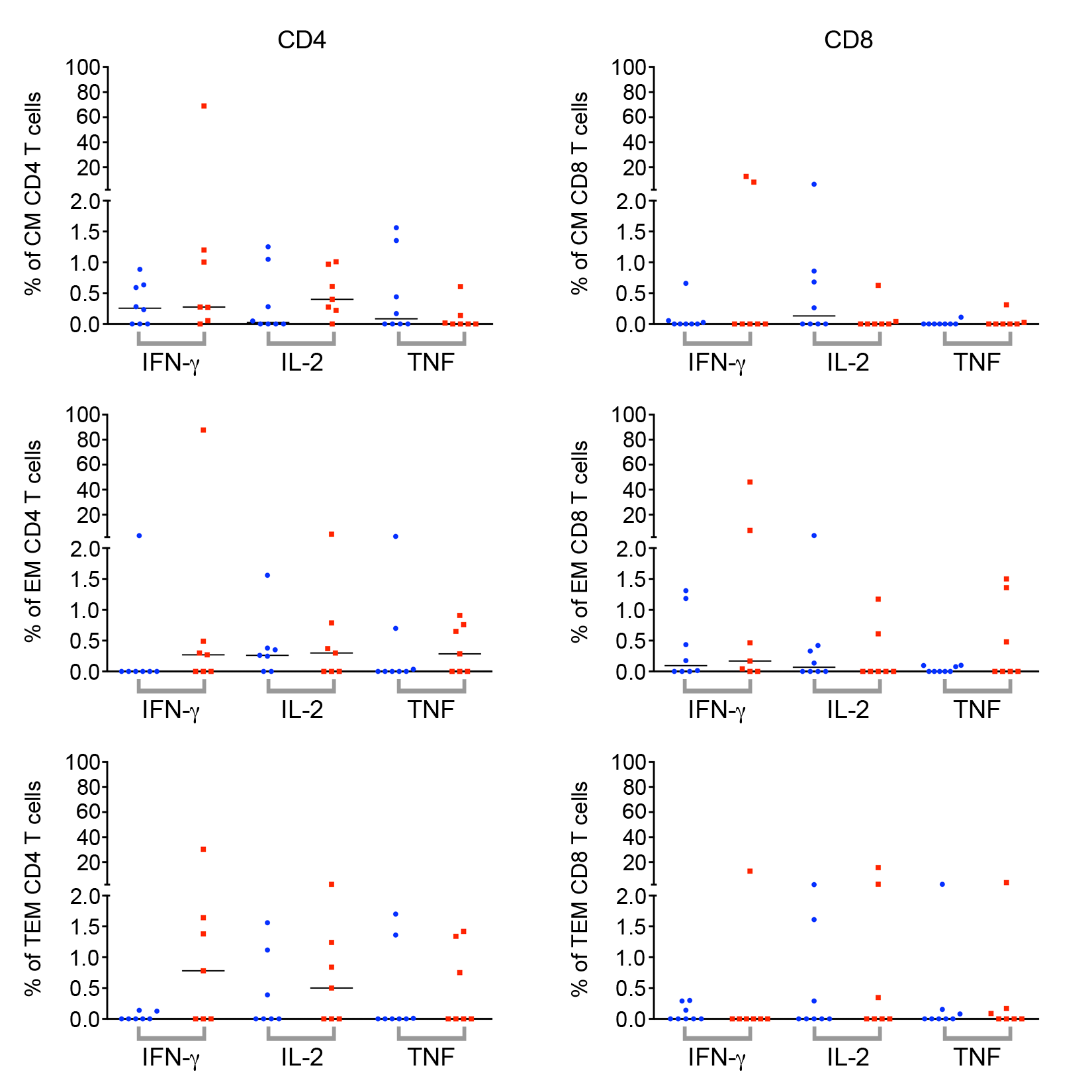

Supplement: S10 Fig — Mycobacterial antigen (ESAT6 and CFP10) specific production of single cytokine staining of IFN-γ, IL-2, and TNF was determined from central memory (CD27+CD45Ra -), effector memory (CD27-CD45Ra-), and terminal effector (CD27-CD45Ra+) populations of CD4 and CD8 T cells. There were no differences in cytokine expression between high and low risk animals. No differences were observed between high and low risk animals. (TIF) [file ppat.1005739.s010.tif]

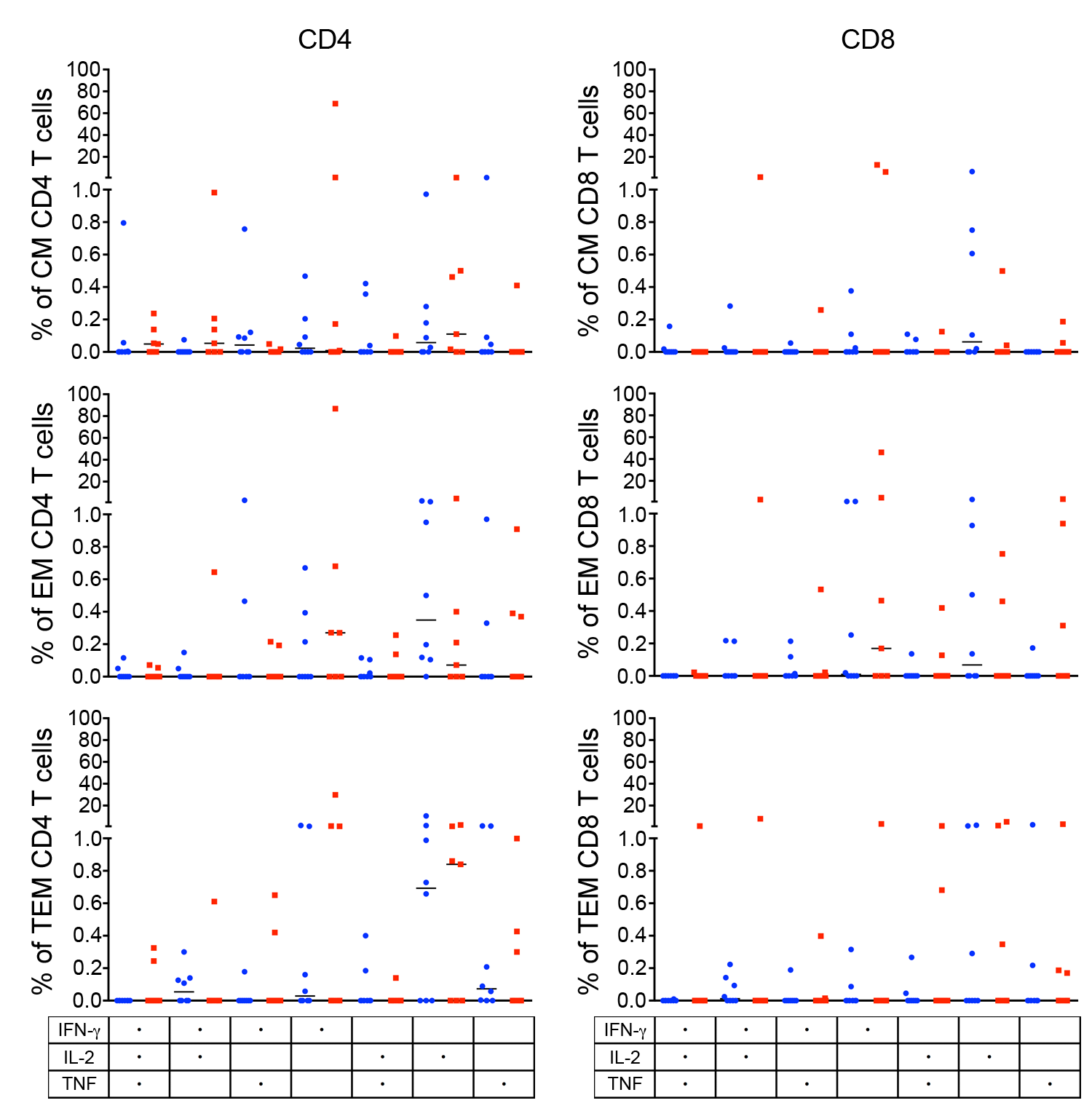

Supplement: S11 Fig — Polyfunctional cytokine (IFN-γ, IL-2, and TNF) staining of mycobacterial antigen (ESAT6 and CFP10) specific production T cells was examined among central memory (CD27+CD45Ra -), effector memory (CD27-CD45Ra-), and terminal effector (CD27-CD45Ra+) populations of CD4 and CD8 T cells. No differences were observed between high and low risk animals. (TIF) [file ppat.1005739.s011.tif]

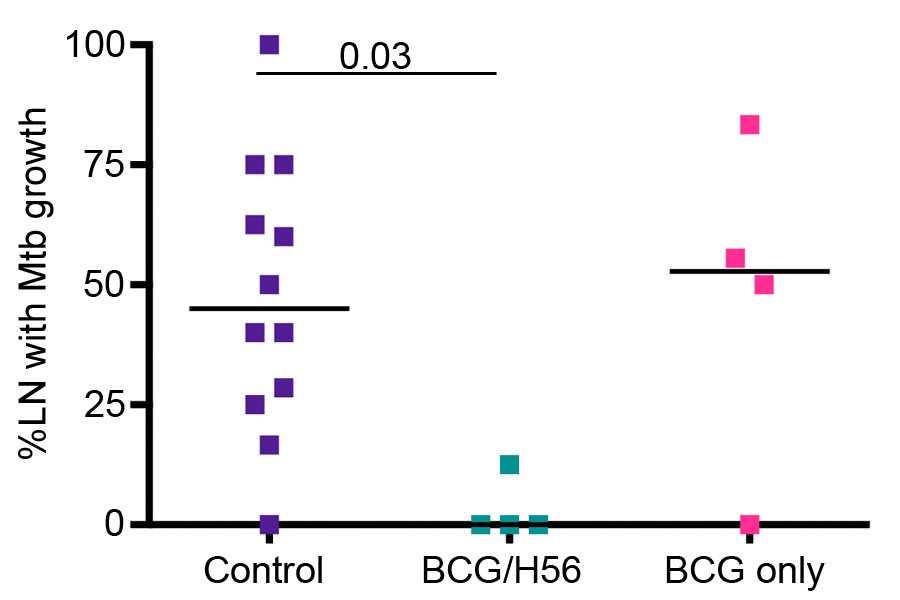

Supplement: S12 Fig — In a previously published vaccine trial [16], macaques were vaccinated with either BCG, a combination of BCG and H56 or were unvaccinated. Latent animals from each group were then treated with anti-TNF antibody; 3 of 4 BCG-vaccinated animals reactivated, similar to latent controls in that study while 0 of 4 BCG/H56 vaccinated animals reactivated. Here, the frequency of MLN with growth of M. tuberculosis was analyzed from those animals. The BCG/H56 animals had a significantly lower proportion of Mtb-positive MLN than the control animals. The indicated p-value is derived from Kruskal Wallis with Dunn’s multiple comparison. (TIF) [file ppat.1005739.s012.tif]
